# Push–Pull Mechanism of Attention and Emotion in Children with Attention Deficit Hyperactivity Disorder

**DOI:** 10.3390/jcm13144206

**Published:** 2024-07-18

**Authors:** Ji-Hyun Song, So-Yeon Kim

**Affiliations:** Department of Psychology, Duksung Women’s University, Seoul 01369, Republic of Korea; jyeony827@gmail.com

**Keywords:** attention deficit hyperactivity disorder, attentional orienting, attentional bias, trait anxiety, anxiety levels

## Abstract

**Background/Objectives**: While deficits in executive attention and alerting systems in children with attention deficit hyperactivity disorder (ADHD) are well-documented, findings regarding orienting attention in ADHD have been inconsistent. The current study investigated the mechanism of attentional orienting in children with ADHD by examining their attentional bias towards threatening stimuli. Furthermore, we explored the modulating role of anxiety levels in ADHD on this attentional bias. **Methods**: In Experiment 1, 20 children with ADHD and 26 typically developing children (TDC) performed a continuous performance task that included task-irrelevant distractions consisting of angry faces and neutral places. In Experiment 2, 21 children with ADHD and 25 TDC performed the same task, but with angry and neutral faces as distractors. To measure children’s anxiety levels, the State-Trait Anxiety Inventory was administered before each experiment. **Results**: In Experiment 1, results revealed no attentional bias effects in children with ADHD, whereas TDC exhibited attentional capture effects by both types of distractors. However, in Experiment 2, ADHD children demonstrated an attentional bias towards angry faces, which revealed a significant positive correlation with their trait anxiety levels (*r* = 0.61, *p* < 0.05). Further analyses combining all ADHD children showed that trait anxiety levels in Experiment 2 were significantly higher than those in Experiment 1. Finally, a significant positive correlation was found between anxiety levels and attentional bias towards angry faces in all ADHD children (*r* = 0.36, *p* < 0.01). **Conclusions**: Children with ADHD exhibited atypical attentional-orienting effects to threats, and their levels of trait anxiety appeared to modulate such attentional-orienting mechanisms.

## 1. Introduction

Attention deficit hyperactivity disorder (ADHD) is a neurodevelopmental disorder characterized by symptoms of inattention, hyperactivity, and impulsivity [1]. Prevalence studies suggest that approximately 10% of children in the general population are diagnosed with ADHD, with higher rates observed in boys compared to girls [2,3]. Central to ADHD is a deficit in attentional functions, which play a crucial role in efficiently selecting relevant information in one’s surroundings to achieve current goals [4].

Posner delineated three core attentional networks: alerting, orienting, and executive control networks [5,6]. The alerting network maintains vigilance, the orienting network facilitates rapid shifting of attention, and the executive control network governs selective attention and conflict resolution [5]. While deficits in executive control and alerting networks have been consistently reported in children with ADHD [7,8], the status of the orienting network remains less clear [9].

Specifically, some studies demonstrated intact functions of orienting in children with ADHD [7,8,10]. However, other studies reported abnormal functions of attentional-orienting mechanisms in children with ADHD. For instance, McDonald and colleagues [11] found that children with ADHD showed difficulty disengaging attention from an invalidly cued location, suggesting deficits in attentional disengagement. Additionally, Ortega and colleagues [12] reported slower response times and lower accuracy in children with ADHD compared to typically developing children (TDC) when valid cues were provided before the target appearance, along with abnormal event-related potential (ERP) components associated with attentional orienting in ADHD. These discrepancies underscore the need for further investigation into attentional-orienting functions in ADHD, especially considering its implications for cognitive and behavioral outcomes.

In addition to the major deficits in attentional functions, ADHD is also associated with deficits in socio-emotional functions, including difficulties in recognizing negative facial emotions [13,14,15]. Faces play a critical role in social interactions, and the ability to process facial expressions develops during late childhood and early adolescence [16]. Notably, faces can involuntarily capture attention, leading to attentional biases, particularly toward threatening facial stimuli [17,18].

Previous research on attentional bias has predominantly focused on attentional bias towards threatening stimuli in neurotypical individuals and those with anxiety disorders [19,20,21]. For example, Kim et al. [21] found that individuals with social anxiety disorder exhibited heightened attentional bias towards angry faces at both behavioral and neural levels. Moreover, a recent study investigated the influence of anxiety levels on attentional bias in neurotypical adults and adolescents, revealing that while the onset of abrupt distractors automatically captured attention in adults irrespective of distractor type, only angry face distractors affected reaction times in adolescents [22]. Additionally, this study demonstrated a positive correlation between levels of state anxiety and attentional capture in adults but not adolescents, suggesting the significance of anxiety levels in attentional bias towards emotional stimuli, particularly in developing populations.

However, research specifically addressing attentional bias towards facial stimuli in children with ADHD is scarce and often overlooks an individual’s anxiety levels [23,24]. However, about 25–30% of children with ADHD are accompanied by anxiety symptoms [25]. High levels of anxiety have been reported to exacerbate symptoms of ADHD and impair emotional interference control, leading to difficulties in handling emotional stimuli [26,27]. Particularly, ADHD children with high trait anxiety are susceptible to external stimuli due to prevalent inattentive symptoms, resulting in deficits in sustained attention [28,29]. Thus, investigating the relationship between attentional bias toward threatening stimuli and anxiety levels in children with ADHD is crucial.

Thus, the present study aimed to address this gap by investigating attentional bias towards emotional facial stimuli in children with ADHD and its relationship with anxiety levels, comparing them with TD children. By employing the paradigm introduced by Parks et al. [18], this study examined attentional bias patterns in response to emotional facial stimuli in children with ADHD. Building upon the design of a previous study [18], our research comprised two experiments. In Experiment 1, we utilized angry faces or non-emotional place images as distractors to explore potential attentional capture and holding effects in both TD and ADHD children, modulated by their anxiety levels. In Experiment 2, we employed both angry or neutral faces as distractors to investigate whether children with ADHD would demonstrate attentional capture or holding effects toward task-irrelevant facial stimuli within the context of all facial distractors. Additionally, we examined the effects of anxiety levels on attentional bias effects toward faces in both TD and ADHD children. In two experiments, we investigated whether children with ADHD exhibit atypical attentional-orienting functions, specifically by lacking attentional capture effects towards threatening stimuli. Additionally, we examined whether these atypical orienting functions in ADHD are modulated by anxiety levels. Our research questions and hypotheses are as follows:

Research Questions:

1. Do children with ADHD exhibit atypical attentional-orienting functions, indicated by a lack of attentional capture effects towards threatening stimuli?
2. Are the atypical orienting functions in children with ADHD modulated by their anxiety levels?

Hypotheses:

**Hypothesis** **1.** *Children with ADHD will display deficits in attentional bias towards emotional facial stimuli compared to TDC.*

**Hypothesis** **2.** *Higher levels of trait anxiety in children with ADHD will be positively associated with an attentional bias towards threatening stimuli.*

## 2. Experiment 1

### 2.1. Materials and Methods

#### 2.1.1. Participants

Twenty children with ADHD (aged 11–15 years, 16 boys, 4 girls) and 26 age-matched typically developing children (TDC; aged 11–15 years, 16 boys, 10 girls) were recruited for this experiment (Table 1). Children with ADHD were diagnosed according to DSM-5 criteria (19 inattentive types and 1 combined type) and were recruited from child mental health and treatment centers in Seoul, Gyeonggi-do, and Chungcheongnam-do in Republic of Korea. Typically, developing children (TDC) were recruited from public schools in Seoul and Gyeonggi-do, Republic of Korea. Parent reports were used to screen for neurodevelopmental or psychiatric conditions in TDC, and none reported any current or past psychiatric conditions. All participants were right-handed, had full-scale IQs (FSIQ) above 80, and had normal or corrected-to-normal vision. Ethical approval for the study protocols and procedures was obtained from the university’s internal review board.

#### 2.1.2. Continuous Performance Task (CPT)

The attention task was created using Presentation version 18.1 (Neurobehavioral Systems) and displayed on a 14-inch monitor with an AMD quad-core laptop. The task replicated the design used by Parks et al. [18], a modified version of the Continuous Performance Task (CPT) developed by Kim and Hopfinger [4] to study attention mechanisms based on stimulus characteristics. As in the previous study [18,22], we chose face and place stimuli as distractors in Experiment 1. Specifically, facial stimuli were selected from the Korean Facial Expressions of Emotion (KOFEE) [30], consisting of eight angry face stimuli (4 females and 4 males). Angry face stimuli were chosen as distractors because they can evoke a sense of threat in participants. To serve as control stimuli with neutral emotional value, eight-place stimuli were also selected as distractors. These place stimuli were chosen from the stimulus set used in Parks et al. [18]. All stimuli were converted to grayscale images.

To perform the task, participants were instructed to fixate on a central point while distractors (either an angry face or a place with a visual angle of 8°37 × 8°37) appeared for 4 s in the center of the screen. The target stimulus, a red “T” (5°88 × 5°88), appeared at the upper right of the screen, overlapping with a black cross. Participants were instructed to respond as quickly and accurately as possible to the direction of the red “T” while ignoring the distractors.

In this task, participants were required to maintain fixation on the central point and disregard the distractor stimuli while responding to the direction of the red “T” (the target). If the target stimulus appeared vertically or horizontally (0°, 90°, 180°, 270°), participants pressed the left mouse button with their right hand. If the target stimulus appeared diagonally (45°, 135°, 225°, 315°), participants pressed the right mouse button with their right hand.

The task involved two types of conditions: distractor type and time condition. The distractor type had two levels: angry face and place. The time condition included five levels: T1, T2, T3, T4, and TBaseline (TB), representing the target presentation timing. T1 indicated the target presented concurrently with the distractor for 1000 ms, T2 indicated the target presented 1000 ms after T1, T3 indicated the target presented 1000 ms after T2, and T4 indicated the target presented 1000 ms after T3. TB represented trials where only the target was presented without distractors and was randomly presented within four conditions lasting 3000 to 6000 ms.

**Figure 1:**
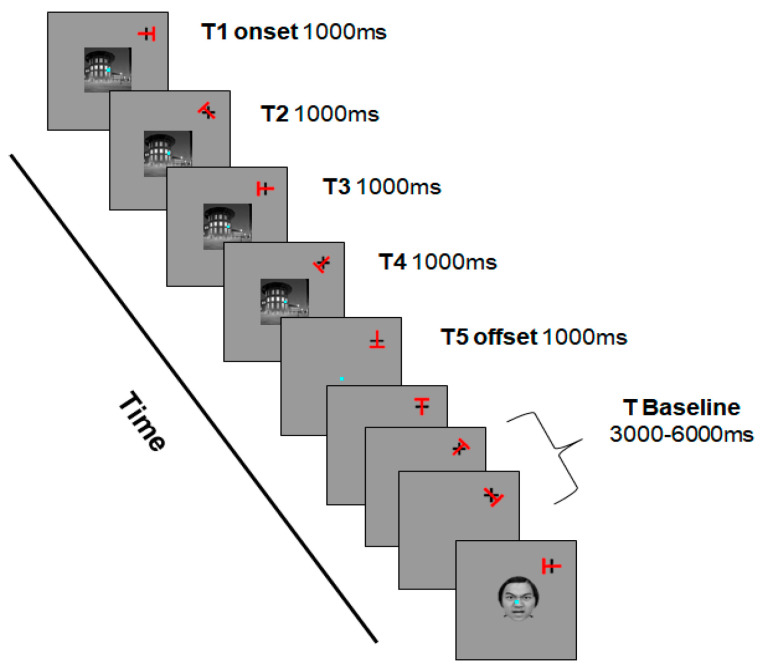
The attention task paradigm of Experiment 1.

In accordance with the methodology outlined by Parks et al. [18], attentional capture was defined as the slow reaction time to the target at T1, while attentional holding referred to the slow response time to the target from T2 to T4. A visual representation of the task is depicted in Figure 1.

Before performing the main CPT, all participants engaged in a passive viewing task designed to expose them to the stimuli used in the primary task, thereby controlling possible attentional capture effects by novel stimuli. This task involved the random presentation of 16 face and place stimuli, each shown twice for 2000 ms, lasting approximately 2 min. Subsequently, to familiarize participants with the task procedure, a practice session was conducted using the same task format, lasting approximately 1 min and 10 s. The main task comprised four blocks, with each block consisting of 204 trials, resulting in a total of 816 trials and lasting approximately 16 min and 20 s. The entire experimental session lasted approximately 20 min.

#### 2.1.3. State-Trait Anxiety Inventory: STAI-X I, II

The State-Trait Anxiety Inventory (STAI-X I, II) [31] was utilized in this study to assess levels of state anxiety and trait anxiety. Each anxiety scale comprised 20 items, rated on a scale from 1 to 4. The state anxiety scale included questions such as “How do you feel at this moment?” while the trait anxiety scale included questions like “How do you generally feel in your daily life?” Scores ranged from 20 to 80, with higher scores indicating higher levels of state and trait anxiety. The reliability and validity of the Korean translation of the STAI-X have been reported to be sufficient (test-retest reliability: *r* = 0.69 for the STAI-X I and *r* = 0.75 for the STAI-X II; Cronbach’s alpha = 0.88 for the STAI-X I and Cronbach’s alpha = 0.83 for the STAI-X II) [32].

#### 2.1.4. Procedures

The tasks were conducted in a quiet environment free from noise distractions. Prior to the experiment, all participants and caregivers provided informed consent, and participants completed the STAI-X I and II questionnaires. Participants then received detailed instructions and explanations regarding the experiment and commenced the task once it was confirmed that they thoroughly understood the instructions. Participants completed the task while seated approximately 60 cm away from the monitor. Upon completion of the task, participants who met the requirements received a 5-dollar gift certificate as compensation. The total duration of the experiment was approximately 40 min, which included 5 min for completing the consent forms, 15 min for completing the STAI-X I, II questionnaire, and 20 min for completing the attention task.

#### 2.1.5. Statistical Analysis

All the data in this study were analyzed using SPSS version 21.0. Reaction times (RTs) less than 150 ms and greater than 1150 ms in the attention task were excluded from the analysis based on previous research [4,18,21,22]. The exclusion was necessary because an RT of 150 ms is too fast for attention to be properly directed in the current task paradigm [4,18]. Furthermore, the target in the current task changed its direction every 1000 ms; thus, it is unclear which trial corresponds to an RT of greater than 1150 ms [4,18,21,22]. For both accuracy and RTs, individuals with mean data 3 standard deviations away from the group average were determined as outliers and excluded from the final analyses. Initial screening analyses revealed that no child in our study met this criterion for being an outlier.

First, descriptive statistics were computed to determine the mean age, state anxiety, trait anxiety levels, and mean RTs for the target. Subsequently, repeated measures ANOVAs were conducted with factors including distractor type (angry face, place), time order (T1, T2, T3, T4, TB), and group (ADHD, TDC). In this context, T1 represented performance on the target presented simultaneously with the distractor, T2 indicated the target following T1, T3 indicated the target following T2, and T4 indicated the target following T3. Partial eta squared (*η*^2^) was used to measure the effect size (small effect size: 0.01; medium effect size: 0.06; large effect size: 0.14) [33,34]. For significant interaction effects, post hoc analyses utilized paired *t*-tests with the Benjamini–Hochberg correction [35] to compare RTs of time order concerning the type of distractors relative to TB. Finally, Pearson correlation analysis was conducted to examine the relationship between the distractor effects and the levels of state and trait anxiety.

### 2.2. Results

**Figure 2:**
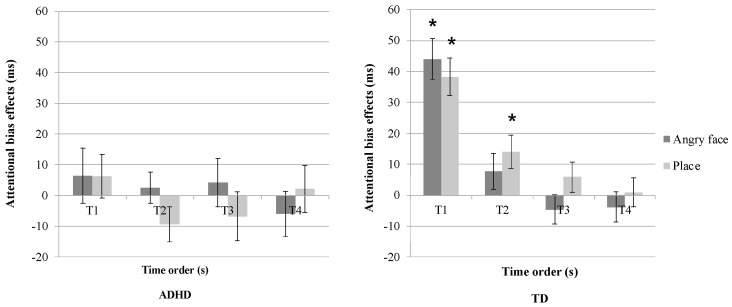
Attentional bias effect on the distractor type and time order in Experiment 1 (* indicates a significant effect with *p* < 0.05).

First, a three-way mixed ANOVA (2 × 5 × 2) was conducted to explore attentional bias effects based on distractor type (angry face, place), time order (T1, T2, T3, T4, TB), and group (ADHD, TDC) (Table 2, Figure 2). The analysis revealed a significant main effect of time order (*F*(2.98, 131.10) = 18.67, *p* < 0.001, *η*^2^ = 0.30) and a significant interaction effect between time order and group (*F*(2.98, 131.10) = 9.20, *p* < 0.001, *η*^2^ = 0.17). Post hoc paired *t*-tests with Benjamini–Hochberg correction [35] were conducted to elucidate the attentional bias across time orders within each group.

The results indicated differential attentional bias effects between ADHD and TD groups across time orders for each distractor type. Specifically, the ADHD group did not exhibit a significant attentional capture effect, regardless of distractor type (all *p*s > 0.05). In contrast, TD children exhibited attentional capture effects for both angry face and place distractors (angry face T1: *t*(25) = 6.65, *p* < 0.001; place T1: *t*(25) = 6.33, *p* < 0.001), with no attentional holding effect for angry face (all *p*s > 0.05). However, a significant attentional holding effect was observed for place distractors at T2 (*t*(25) = 2.62, *p* < 0.05) in the TD group.

Subsequently, correlation analyses were conducted to examine the relationships between anxiety levels and attentional bias effects within each group. Specifically, correlations were assessed between the distraction effects of angry face or place stimuli and state-trait anxiety scores for both groups. The results indicated no statistically significant correlation between distraction effects caused by either an angry face or place and state or trait anxiety levels in either TD or ADHD children.

## 3. Experiment 2

### 3.1. Materials and Methods

#### 3.1.1. Participants

A total of 21 children aged 11 to 15 diagnosed with ADHD (17 boys and 4 girls), based on DSM-5 criteria, were recruited from child mental health facilities and treatment centers located in Seoul, Gyeonggi-do, and Chungcheongnam-do, Republic of Korea. Among the subtypes of ADHD, 12 were classified as inattentive, and 9 were classified as combined. Additionally, 25 age-matched TD children (16 boys and 9 girls) were recruited from general schools in Seoul and Gyeonngi-do, Republic of Korea. One TD boy was excluded from the final analysis due to RTs exceeding three standard deviations from the mean. Demographic information for the participants included in the final analysis is presented in Table 3. All participants had normal or corrected-to-normal vision and were right-handed. The study procedures were approved by the Ethics Committee of the University.

#### 3.1.2. Tasks and Procedures

**Figure 3:**
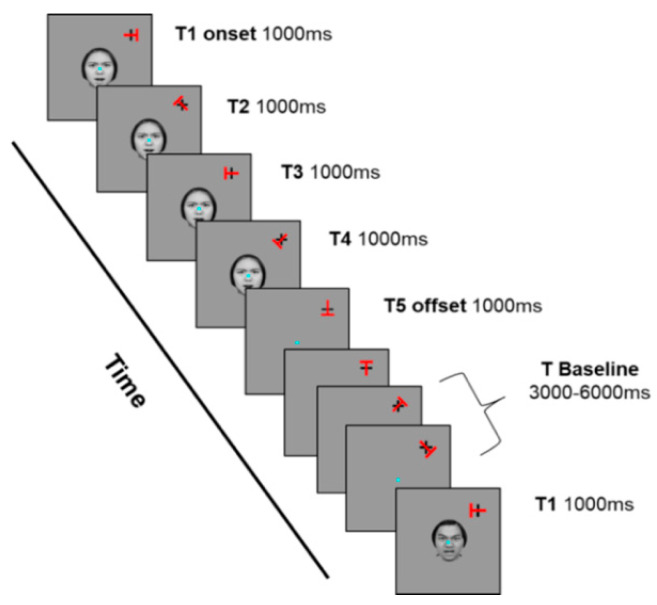
The attention task paradigm of Experiment 2.

The Continuous Performance Task (CPT) was conducted using the same computer setup as in Experiment 1. Experiment 2 utilized the same CPT as Experiment 1 with one modification: instead of non-emotional place images, Experiment 2 included non-emotional neutral face stimuli along with angry face stimuli (Figure 3). Each set of face stimuli consisted of 4 women and 4 men, totaling 16 face stimuli. Apart from the types of distractors, all procedures, anxiety questionnaires, and statistical analyses remained identical to those in Experiment 1.

### 3.2. Results

**Figure 4:**
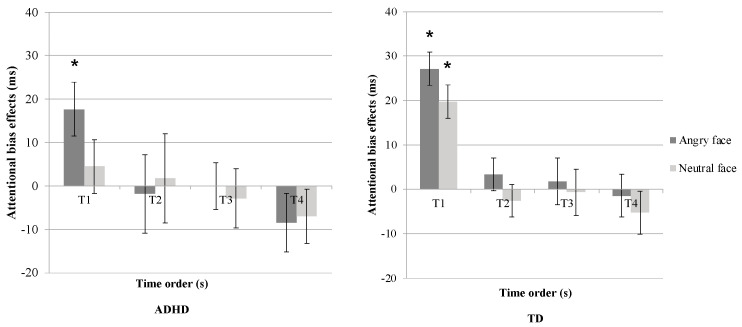
Attentional bias effect on the distractor type and time order in Experiment 2. (* indicates a significant effect with *p* < 0.05).

A three-way mixed ANOVA (2 (Group: ADHD/TDC) × 2 (distractor type: angry/neutral face) × 5 (time order: T1, T2, T3, T4, TB) conducted on the RT data in the CPT revealed a trend-level significance for the main effect of distractor type (*F*(1, 44) = 3.91, *p* = 0.054, *η*^2^ = 0.08), indicating a potential difference in attentional bias between angry and neutral face stimuli (Table 4). Moreover, a significant main effect of time order was observed (*F*(2.97, 130.85) = 11.83, *p* < 0.001, *η*^2^ = 0.21), suggesting variations in RTs across different time points. However, no significant interaction effect was found. Post hoc paired *t*-tests with Benjamini–Hochberg correction were conducted to explore the main effects of distractor type and time order (Figure 4).

Results indicated that ADHD children and TD children demonstrated differing patterns depending on the distractor type and time order. Specifically, ADHD children exhibited a significant attentional capture effect when the distractor was an angry face (T1; *t*(20) = 2.85, *p* < 0.05), whereas no significant attentional capture effect was observed with neutral face stimuli. Conversely, TD children demonstrated attentional capture effects for both angry and neutral face stimuli (T1; angry face: *t*(24) = 7.22, *p* < 0.001, neutral face: *t*(24) = 4.95, *p* < 0.001). In both groups, no significant attentional holding effects were observed for either distractor type (all *p*s > 0.5).

**Figure 5:**
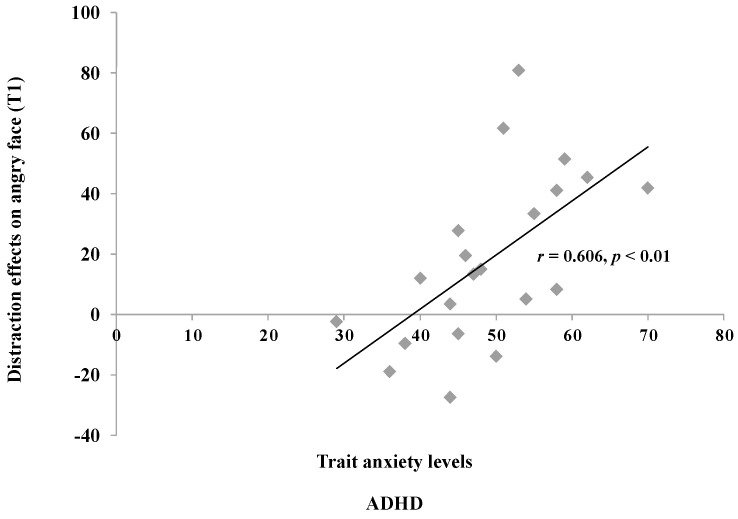
Correlation between trait anxiety and distraction effects on angry face in ADHD children in Experiment 2.

Correlation analyses were subsequently performed to examine the relationships between attentional bias scores and anxiety levels within each group. The results indicated a significant positive correlation between distraction effects induced by angry face stimuli and trait anxiety levels in ADHD children (r = 0.61, *p* < 0.05), suggesting that higher trait anxiety levels were associated with greater attentional capture effects by angry face stimuli in this group (Figure 5). However, no statistically significant correlations were found between state-trait anxiety levels and distraction effects in the TD group.

**Figure 6:**
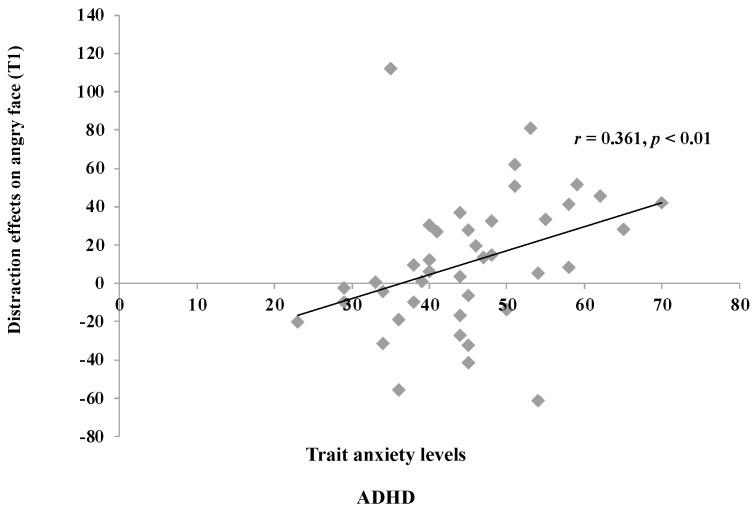
Correlation between trait anxiety and distraction effects on the angry face in all ADHD children.

Of particular note, we observed a statistically significant correlation between trait anxiety levels and attentional capture scores solely in children with ADHD in Experiment 2. To thoroughly elucidate the disparity between the findings of the two experiments, we conducted an independent *t*-test to examine whether there was a difference in state and/or trait anxiety levels among ADHD children across the two experiments. The results revealed no significant disparity in state anxiety levels between the two experiments, whereas a significant difference was found in trait anxiety levels (*t*(39) = −2.80, *p* < 0.01). Specifically, the trait anxiety levels of ADHD children in Experiment 2 were significantly higher than those in Experiment 1. Furthermore, a statistically significant correlation was observed between attentional capture effects by angry face distractors and trait anxiety levels among all ADHD children across Experiments 1 and 2 (*r* = 0.361, *p* < 0.01; Figure 6).

Overall, our findings collectively suggest that ADHD children exhibit distinct attentional bias patterns compared to their TD counterparts, particularly in response to emotional facial stimuli. Additionally, our results indicate that trait anxiety may exert a differential influence on attentional processes in individuals with ADHD.

## 4. Discussion

Our study aimed to explore attentional bias patterns towards emotional facial stimuli in children with ADHD and TD. The findings from Experiment 1, which utilized angry face and place stimuli as distractors, indicated that ADHD children did not demonstrate significant attentional capture or attentional holding effects for either angry face or place stimuli. Conversely, TD children exhibited a significant attentional capture effect for both distractors, along with an attentional holding effect for place stimuli at T2.

In Experiment 2, which involved angry and neutral face stimuli as distractors, we found a significant attentional capture effect only for angry face distractors in children with ADHD. TD children, however, showed an attentional capture effect for all distractors but no attentional holding effect for any distractors. These findings in TD children are consistent with previous studies using the same task paradigm, where attentional capture effects but not holding effects were reported when all the distractors were from the same category (e.g., face) both in TD children [22] and in neurotypical adults [18,21]. Therefore, our findings suggest that children with ADHD exhibit atypical attentional bias patterns, characterized by reduced attentional capture effects by face distractors, with the exception of face stimuli displaying angry expressions.

Interestingly, children with ADHD in our study only exhibited attentional capture effects in response to angry face distractors in Experiment 2, while they did not display significant attentional bias effects towards neutral faces, neutral places, or angry face distractors in Experiment 1. These results prompt inquiries regarding the influence of anxiety levels on attentional orientation processes in children with ADHD. Our correlation analyses revealed that while no statistically significant correlation was found between distraction effects caused by angry face stimuli and trait anxiety levels in ADHD children in Experiment 1, a significant positive correlation was observed in Experiment 2. Conversely, no statistically significant correlations were found between distraction effects on angry or neutral face stimuli and state or trait anxiety levels in TD children across both experiments.

Subsequent analyses comparing anxiety levels in ADHD children between Experiment 1 and 2 revealed significantly higher anxiety levels in ADHD children in Experiment 2 than those in Experiment 1. Finally, correlation analyses including all ADHD children in our study also showed a significant positive correlation between trait anxiety levels and attentional bias scores to angry face distractors.

Overall, our findings suggest that anxiety levels in ADHD children, rather than attentional functions per se, play a crucial role in attentional orienting to emotional face stimuli. In other words, our findings indicate atypical attentional-orienting functions in children with ADHD, with anxiety levels modulating attentional-orienting functions toward angry face distractors.

In both experiments, TD children exhibited an initial attentional capture effect for all distractors, consistent with previous studies demonstrating such responses to sudden emergent stimuli [4,18,21,22]. However, unlike previous findings [18], our study revealed that TD children displayed attentional capture and holding effects for place stimuli rather than face stimuli when the context of distractors was inconsistent (e.g., place and face). These discrepancies from prior research may be attributed to the type of place stimuli employed in our study. Specifically, the place stimuli we utilized reflected foreign cultures, potentially differing culturally from places in Republic of Korea. Since human cultural contexts can influence cognitive and attentional functions [36], participants in our study may have exhibited different attentional patterns compared to those in previous studies. Thus, future research should consider modifying place stimuli to align with the cultural context.

In contrast, children with ADHD did not demonstrate attentional capture or attentional holding effects for either angry face or place stimuli in Experiment 1. This finding is consistent with previous research indicating deficits in covert orienting attention in individuals with ADHD [11,12,37]. Parks et al. [18] reported that unconscious attentional capture occurred irrespective of stimulus type when stimuli suddenly appeared in a typically developing population. However, given the attentional-orienting difficulties observed in ADHD children, their pattern of attentional capture effects may differ from that of TD children. Thus, ADHD children did not exhibit attentional capture for either angry face or place stimuli.

However, Experiment 2 yielded contrasting results, with ADHD children showing an initial attentional bias effect towards angry face distractors. This finding deviates from previous studies [23,24], which reported no attentional bias towards negative emotional stimuli in ADHD children. This discrepancy may be explained by differences in anxiety levels among children with ADHD. Although the same angry face stimuli were used in both experiments, varying anxiety levels were observed among ADHD children in Experiments 1 and 2. Specifically, significant differences in trait anxiety levels were evident, with ADHD children exhibiting higher trait anxiety scores in Experiment 2 compared to those in Experiment 1. Moreover, all ADHD children across both experiments demonstrated a statistically significant correlation between trait anxiety levels and distraction effects caused by angry face stimuli. These findings suggest that attentional bias towards negative facial stimuli in ADHD children may be affected by their anxiety levels.

In previous studies [23,24], where anxiety levels were not measured in ADHD children, no attentional bias towards negative facial stimuli was observed. Specifically, the face-dot probe task used in previous research presented a cue to the target stimuli by presenting a probe, which appeared at the same or opposite site after the face stimuli [24]. Therefore, attentional bias effects may not be consistently reported because facial emotional stimuli and target stimuli are related to each other. However, our study presented task-irrelevant distractors, allowing us to confirm the impact of facial emotions on attention as completely unrelated distractors. Additionally, the previous study did not compare ADHD children with TD children [24]. Another previous study using an eye-tracking paradigm did not consider cover attention, which is attention without eye movements [23]. In contrast, our study was able to measure covert attention by orienting attention without eye movements.

Furthermore, our examination of anxiety levels revealed that ADHD children with high trait anxiety may exhibit an attentional bias towards negative facial emotions. This is consistent with studies examining attentional bias for threats among individuals with high trait anxiety or social anxiety [19,20,21,26,27,38]. Specifically, individuals with higher anxiety levels are more likely to allocate attention to stimuli with negative emotions than those without emotions [19,20,38]. Additionally, high levels of trait anxiety are associated with susceptibility to threat-related emotional stimuli [26,27].

Overall, the results from Experiments 1 and 2 suggest that ADHD children exhibit deficits in orienting attention, thereby failing to appropriately allocate attention to stimuli. Consequently, ADHD children may lack early attentional capture not only for angry and neutral facial stimuli but also for place stimuli. Furthermore, the observed attentional bias towards angry face distractors in ADHD children with high anxiety levels suggests that psychological characteristics, such as anxiety, may prompt attentional allocation to threatening facial stimuli rather than compensating for orienting attention deficits [20,38].

In TD children, a greater attentional capture effect was noted for angry face distractors compared to neutral face distractors, given their sensitivity to salient threatening cues in the environment [39]. Specifically, angry face stimuli may evoke heightened sensitivity among TDC, as they serve as social cues directly communicating threats in the environment [40,41,42]. Furthermore, no statistically significant correlations were found between distraction effects on angry face stimuli and state/trait anxiety levels in TDC. These findings align with previous studies [22,43] that reported no relationship between distraction effects on negative emotional stimuli and anxiety levels. Thus, the initial attentional bias toward negative face distractors in TDC appears to be a developmental characteristic sensitive to threatening stimuli rather than anxiety levels.

Several limitations of our study are worth mentioning. Firstly, there was an imbalance in the gender ratio among participants, with more boys than girls in both groups. Although the gender ratio was statistically insignificant between the ADHD and the TD groups in both experiments, the proportion of girls was slightly lower in the ADHD group than in the TD group. This gender bias in recruitment aligns with the higher prevalence of ADHD among boys compared to girls [2]. While the reasons for the imbalanced prevalence rates between genders in ADHD are still unclear, recent research has discussed differences in parent perceptions of ADHD behaviors in girls and the necessity of assessing additional behavioral and emotional problems in girls [44]. Although recent studies have indicated no significant gender differences in the cognitive functioning of children with ADHD [45,46], achieving gender balance in future studies can be beneficial for a comprehensive understanding of ADHD. Second, the potential impact of cultural differences on the stimuli in Experiment 1 should be considered. As discussed earlier, cultural contexts can influence cognitive and attentional functions [36], and our use of pictures of foreign places as distractors might have contributed to the different attentional patterns observed in our study compared to previous research. Future studies should consider modifying place stimuli to better align with the cultural context of the participants, ensuring that the stimuli are culturally relevant and recognizable. This adjustment could provide a more accurate assessment of attentional biases and reduce potential confounding effects related to cultural differences. Third, our study identified a significant positive correlation between trait anxiety levels and attentional bias towards threatening facial stimuli in children with ADHD. To further elucidate the relationship between attentional bias and varying anxiety levels, future research should differentiate between children with ADHD who exhibit high anxiety levels and those with low anxiety levels. Additionally, incorporating a variety of anxiety scales to measure levels of anxiety in participants would be valuable. Lastly, our study did not control for eye movements and employed adult face stimuli as distractors. Future studies could utilize eye-tracking technology to more accurately investigate the attentional bias patterns toward emotional stimuli in children with ADHD. Considering that individuals are more accurate in perceiving face stimuli of their own age group [47], employing child face stimuli for child participants could enhance the precision of measuring attentional mechanisms.

Despite these limitations, our study is significant as the first to investigate attentional bias towards emotional face stimuli over time in children with ADHD, revealing that orienting attentional deficits in ADHD are modulated by their anxiety levels. Ultimately, our findings suggest that anxiety may affect attentional orienting in children with ADHD. Moreover, our findings indicate that children with ADHD may struggle to allocate attention not only to emotional face stimuli but also to place stimuli, likely due to difficulties in orienting attention. While previous research has predominantly focused on improving executive and vigilance attention in the treatment and evaluation of ADHD [7,8], our results highlight the importance of addressing deficits in orienting attentional mechanisms in interventions for children with ADHD. Specifically, our results suggest that interventions for children with ADHD should incorporate strategies to manage anxiety, as anxiety levels appear to influence attentional orienting. By addressing anxiety, it may be possible to improve the orienting attention of children with ADHD, thereby enhancing their ability to process both emotional and neutral stimuli effectively. Furthermore, developing specific attention assessment tools that include measures of orienting attention could lead to more targeted and effective treatment plans. These tools could help identify children who are particularly affected by anxiety-related attentional biases, allowing for personalized intervention strategies that address both attentional and emotional regulation needs.

While our research provides novel evidence of attentional-orienting mechanisms in children with ADHD and the modulation effects of an individual’s anxiety levels on the orienting function, it will be worthwhile to explore whether such effects are also found in adults with ADHD. Additionally, future research could enhance our understanding of individuals with ADHD by utilizing various emotional stimuli, such as happy faces/scenes, or unpleasant stimuli, such as disgusting faces/scenes. Finally, future research could employ a broader range of anxiety measures, such as assessments of social anxiety or general anxiety, to elucidate the effects of anxiety on attentional orienting in individuals with ADHD in greater detail.

## Figures and Tables

**Table 1:** Participants’ characteristics included in Experiment 1.

Group	Sex		Age	State Anxiety	Trait Anxiety
	Boy (%)	Girl (%)	M (SD)	M (SD)	M (SD)
ADHD (N = 20)	16 (80)	4 (20)	13.17 (1.27)	39.45 (8.37)	40.90 (9.32)
TDC (N = 26)	16 (62)	10 (38)	13.37 (1.51)	35.88 (7.53)	39.00 (7.71)

**Table 2:** An ANOVA table in Experiment 1.

	*F*	*p*	*η* ^2^
Distractor	*F*(1, 44) = 2.41	0.13	0.05
Time order	*F*(2.98, 131.10) = 18.67	<0.001	0.30
Group	*F*(1, 44) = 1.84	0.18	0.04
Distractor × Group	*F*(1, 44) = 0.51	0.48	0.01
Time order × Group	*F*(2.98, 131.10) = 9.20	<0.001	0.17
Distractor × Time order	*F*(4, 176) = 0.786	0.54	0.02
Distractor × Time order × Group	*F*(3.51, 154.46) = 2.18	0.083	0.05

**Table 3:** Participants’ characteristics included in Experiment 2.

Group	Sex		Age	State Anxiety	Trait Anxiety
	Boy (%)	Girl (%)	M (SD)	M (SD)	M (SD)
ADHD (N = 21)	17 (81)	4 (19)	12.64 (1.29)	38.14 (7.84)	49.14 (9.54)
TDC (N = 25)	16 (64)	9 (36)	12.64 (1.07)	35.24 (5.39)	41.48 (9.14)

**Table 4:** An ANOVA table in Experiment 2.

	*F*	*p*	*η* ^2^
Distractor	*F*(1, 44) = 3.91	0.05	0.08
Time order	*F*(2.97, 130.85) = 11.83	<0.001	0.21
Group	*F*(1, 44) = 0.13	0.72	0.003
Distractor × Group	*F*(1, 44) = 0.97	0.33	0.02
Time order × Group	*F*(4, 176) = 0.997	0.41	0.02
Distractor × Time order	*F*(2.81, 123.63) = 0.75	0.56	0.02
Distractor × Time order × Group	*F*(2.81, 123.63) = 0.38	0.77	0.01

## Data Availability

Individual data are protected and thus are not shareable.

## References

[1] American Psychiatric Association (2013). Diagnostic and Statistical Manual of Mental Disorders (DSM-5®).

[2] Cho S.C., Kim B.N., Kim J.W., Rohde L.A., Hwang J.W., Chungh D.S., Shin M.S., Lyoo I.K., Go B.J., Lee S.E. (2009). Full syndrome and subthreshold attention-deficit/hyperactivity disorder in a Korean community sample: Comorbidity and temperament findings. Eur. Child. Adolesc. Psychiatry.

[3] Polanczyk G.V., Willcut E.G., Salum G.A., Kieling C., Rohde L.A. (2014). ADHD prevalence estimates across three decades: An updated systematic review and meta-regression analysis. Int. J. Epidemiol..

[4] Kim S.-Y., Hopfinger J.B. (2010). Neural basis of visual distraction. J. Cogn. Neurosci..

[5] Posner M.I. (1980). Orienting of attention. Q. J. Exp. Psychol..

[6] Fan J., McCandliss B.D., Sommer T., Raz A., Posner M.I. (2002). Testing the efficiency and independence of attentional networks. J. Cogn. Neurosci..

[7] Oberlin B.G., Alford J.L., Marrocco R.T. (2005). Normal attention orienting but abnormal stimulus alerting and conflict effect in combined subtype of ADHD. Behav. Brain Res..

[8] Mullane J.C., Corkum P.V., Klein R.M., McLaughlin E.N., Lawrence M.A. (2011). Alerting, orienting, and executive attention in children with ADHD. J. Atten. Disord..

[9] Kim S.-Y. (2014). Attentional Functions and Dysfunctions in ADHD. Korean J. Cogn. Biol. Psychol..

[10] Johnson K.A., Robertson I.H., Barry E., Mulligan A., Dáibhis A., Daly M., Watchorn A., Gill M., Bellgrove M.A. (2008). Impaired conflict resolution and alerting in children with ADHD: Evidence from the Attention Network Task (ANT). J. Child Psychol. Psychiatry.

[11] McDonald S., Bennett K.M.B., Chambers H., Castiello U. (1999). Covert orienting and focusing of attention in children with attention deficit hyperactivity disorder. Neuropsychologia.

[12] Ortega R., López V., Carrasco X., Anllo-Vento L., Aboitiz F. (2013). Exogenous orienting of visual-spatial attention in ADHD children. Brain Res..

[13] Wehmeier P.M., Schacht A., Barkley R.A. (2010). Social and emotional impairment in children and adolescents with ADHD and the impact on quality of life. J. Adolesc. Health.

[14] Caillies S., Bertot V., Motte J., Raynaud C., Abely M. (2014). Social cognition in ADHD: Irony understanding and recursive theory of mind. Res. Dev. Disabil..

[15] Dan O., Raz S. (2018). Response patterns to emotional faces among adolescents diagnosed with ADHD. J. Atten. Disord..

[16] Birmingham E., Meixner T., Iarocci G., Kanan C., Smilek D., Tanaka J.W. (2013). The moving window technique: A window into developmental changes in attention during facial emotion recognition. Child Dev..

[17] Langton S.R., Law A.S., Burton A.M., Schweinberger S.R. (2008). Attention capture by faces. Cognition.

[18] Parks E.L., Kim S.-Y., Hopfinger J.B. (2014). The persistence of distraction: A study of attentional biases by fear, faces, and context. Psychon. Bull. Rev..

[19] Mogg K., Bradley B.P. (1998). A cognitive-motivational analysis of anxiety. Behav. Res. Ther..

[20] Mogg K., Bradley B.P., De Bono J., Painter M. (1997). Time course of attentional bias for threat information in non-clinical anxiety. Behav. Res. Ther..

[21] Kim S.-Y., Shin J.E., Lee Y.J., Kim H.N., Choi S.H. (2018). Neural evidence for persistent attentional bias to threats in patients with social anxiety disorder. Soc. Cogn. Affect. Neurosci..

[22] Kim M., Kim J., Kim S.-Y. (2022). Attentional Bias to Emotional Stimuli and Effects of Anxiety on the Bias in Neurotypical Adults and Adolescents. Sci. Emot. Sensib..

[23] Ahmadi M., Judi M., Khorrami A., Mahmoudi-Gharaei J., Tehrani-Doost M. (2011). Initial orientation of attention towards emotional faces in children with attention deficit hyperactivity disorder. Iran. J. Psychiatry.

[24] Weissman A.S., Chu B.C., Reddy L.A., Mohlman J. (2012). Attention mechanisms in children with anxiety disorders and in children with attention deficit hyperactivity disorder: Implications for research and practice. J. Clin. Child Adolesc. Psychol..

[25] Jarrett M.A., Ollendick T.H. (2008). A conceptual review of the comorbidity of attention-deficit/hyperactivity disorder and anxiety: Implications for future research and practice. Clin. Psychol. Rev..

[26] Bishop S.J., Duncan J., Lawrence A.D. (2004). State anxiety modulation of the amygdala response to unattended threat-related stimuli. J. Neurosci..

[27] MacNamara A., Ferri J., Hajcak G. (2011). Working memory load reduces the late positive potential and this effect is attenuated with increasing anxiety. Cogn. Affect. Behav. Neurosci..

[28] Bloemsma J.M., Boer F., Arnold R., Banaschewski T., Faraone S.V., Buitelaar J.K., Faraone S.V., Buitelaar J.K., Sergeant J.A., Rommelse N. (2013). Comorbid anxiety and neurocognitive dysfunctions in children with ADHD. Eur. Child. Adolesc. Psychiatry.

[29] González-Castro P., Rodríguez C., Cueli M., García T., Alvarez-García D. (2015). State, trait anxiety and selective attention differences in Attention Deficit Hyperactivity Disorder (ADHD) subtypes. Int. J. Clin. Health Psychol..

[30] Park J.Y., Oh J.M., Kim S.Y., Lee M.K., Lee C.R., Kim B.R., An S.K. (2011). Korean Facial Expressions of Emotion (KOFEE).

[31] Spielberger C.D., Gorsuch R.L., Lushene R.E. (1970). Manual for the State-Trait Inventory.

[32] Cho S., Choi J. (1989). Development of the Korean form of the State-trait Anxiety Inverntory for Children. Seoul J. Psychiatry.

[33] Richardson J.T.E. (2011). Eta squared and partial eta squared as measures of effect size in educational research. Educ. Res. Rev..

[34] Gonçalves J.R., Sleath B.L., Cerdeira M., Cavaco A.M. (2023). Older people, medication usage and long-term care pharmacists: A retrospective cohort study. Eur. J. Hosp. Pharm..

[35] Benjamini Y., Hochberg Y. (1995). Controlling the False Discovery Rate: A Practical and Powerful Approach to Multiple Testing. J. R. Stat. Soc. Ser. B Stat. Method.

[36] Han S., Northoff G. (2008). Culture-sensitive neural substrates of human cognition: A transcultural neuroimaging approach. Nat. Rev. Neurosci..

[37] Konrad K., Neufang S., Hanisch C., Fink G.R., Herpertz-Dahlmann B. (2006). Dysfunctional attentional networks in children with attention deficit/hyperactivity disorder: Evidence from an event-related functional magnetic resonance imaging study. Biol. Psychiatry.

[38] Fox E., Russo R., Bowles R., Dutton K. (2001). Do threatening stimuli draw or hold visual attention in subclinical anxiety?. J. Exp. Psychol. Gen..

[39] Somerville L.H., Jones R.M., Casey B.J. (2010). A time of change: Behavioral and neural correlates of adolescent sensitivity to appetitive and aversive environmental cues. Brain Cogn..

[40] Berkowitz L. (1990). On the formation and regulation of anger and aggression: A cognitive-neoassociationistic analysis. Am. Psychol..

[41] Adams R.B., Gordon H.L., Baird A.A., Ambady N., Kleck R.E. (2003). Effects of gaze on amygdala sensitivity to anger and fear faces. Science.

[42] Adolphs R., Russell J.A., Tranel D.A. (1999). A role for the human amygdala in recognizing emotional arousal from unpleasant stimuli. Psychol. Sci..

[43] Waters A.M., Lipp O.V., Spence S.H. (2004). Attentional bias toward fear-related stimuli: An investigation with nonselected children and adults and children with anxiety disorders. J. Exp. Child. Psychol..

[44] Mowlem F., Agnew-Blais J., Taylor E., Asherson P. (2019). Do different factors influence whether girls versus boys meet ADHD diagnostic criteria? Sex differences among children with high ADHD symptoms. Psychiatry Res..

[45] Martel M.M. (2013). Individual Differences in Attention Deficit Hyperactivity Disorder Symptoms and Associated Executive Dysfunction and Traits: Sex, Ethnicity, and Family Income. Am. J. Orthopsychiatry.

[46] Skogli E.W., Teicher M.H., Andersen P.N., Hovik K.T., Øie M. (2013). ADHD in girls and boys–gender differences in co-existing symptoms and executive function measures. BMC Psychiatry.

[47] Rhodes M.G., Anastasi J.S. (2012). The own-age bias in face recognition: A meta-analytic and theoretical review. Psychol. Bull..

